# High-dose-rate monotherapy for localised prostate Cancer: A two-implant approach with median 13 years follow-up

**DOI:** 10.1016/j.ctro.2026.101228

**Published:** 2026-07-08

**Authors:** Christopher Best, Valentin Spier, Dimos Baltas, Hanns Ackermann, Claus Rödel, Nikolaos Zamboglou, Nikolaos Tselis

**Affiliations:** a Institute of Medical Microbiology and Infection Control, Goethe University Frankfurt, University Hospital, Frankfurt am Main, Germany; b Department of Radiotherapy and Oncology, Goethe University Frankfurt, University Hospital, Frankfurt am Main, Germany; c Division of Medical Physics, Department of Radiation Oncology, Medical Centre - University of Freiburg, Germany; d Institute for Biostatistics and Mathematical Modelling, Goethe University Frankfurt, University Hospital, Frankfurt am Main, Germany; e Department of Radiation Oncology, German Oncology Centre, Limassol, Cyprus

**Keywords:** Prostate cancer, Brachytherapy, High-dose-rate, monotherapy

## Abstract

**Objectives:**

To report the clinical outcome of High-Dose-Rate (HDR) brachytherapy (BRT) as sole treatment for clinically localised prostate cancer (PCA).

**Methods:**

From March 2004 to January 2008, 351 consecutive patients with clinically localised PCA were treated with HDR monotherapy receiving two BRT implants, separated by 14 days, each delivering two fractions at 9.5 Gy to an intraoperative transrectal ultrasound real-time defined planning treatment volume**.** The present report describes the updated long-term results of this two-implant protocol. Biochemical relapse was defined according to the Phoenix definition and toxicity evaluated using the Common Toxicity Criteria for Adverse Events version 3.

**Results:**

The median follow-up for the current analysis was 158 months with 201 patients eligible for analysis. Out of the initial cohort, 62 patients died during follow-up and 150 were lost to follow-up. The 60-, 120- and 158-months biochemical control and metastasis-free survival rates were 94%, 90%, 86% and 97%, 89%, 83%, respectively. Eight (3.9%) patients developed late Grade 2–3 lower urinary tract symptoms characterized by increased urgency and frequency with no dysuria or incontinence > Grade 2. Four (2%) patients developed late Grade 3 gastrointestinal toxicity. No Grade 4 or higher acute or late toxicities occurred.

**Conclusions:**

Our results confirm HDR-BRT to be a safe and effective monotherapy for organ-confined PCA.

## Highlights

• Two-implant based HDR monotherapy 13 year biochemical control (BC) of 86%.
• Low risk patients 13 year 94 BCE%.
• Intermediate risk patients 13 year 85 BCE%.
• Grade ≥ 2 late gastrointestinal (GI) toxicity 2.5% and late genitourinary (GU) toxicity 12%.
• No Grade 4 late GI/GU toxicity.

## 1 Introduction

Prostate cancer (PCA) is the 2nd most commonly occurring cancer in men and the 4th most common cancer overall [1]. Based on the widespread use of prostate-specific antigen (PSA)-testing, its incidence has risen with an inverse development in staging and risk-stratification in the last decades [2].

There are multiple treatment options for patients with clinically localised disease, including radical prostatectomy, androgen deprivation therapy (ADT), external beam radiotherapy (EBRT) [3], [4], and brachytherapy (BRT) [5], [6], [7], [8], all achieving similar rates of biochemical control (BC) although with differing degrees and types of genitourinary (GU) and gastrointestinal (GI) morbidities [9].

In the absence of phase III comparative efficacy data, however, the optimal management remains controversial and treatment assignment is usually influenced by physician bias and patient preference [10]. Given that multiple trials support an association between dose-escalated radiotherapy (RT) and improved clinical outcomes [11], [12], [13], high-dose-rate (HDR) BRT has proven to optimally exploit the advantage of biological dose escalation through hypofractionation while ensuring superior conformity [14]. At this, the versatility of intratarget dose modulation inherent to afterloading BRT enables dosimetric optimisation which cannot be reproduced even with the most conformal external beam techniques [15].

We previously reported the outcomes of HDR monotherapy in 351 patients with localised PCA at a median follow-up of 59.3 months [16]. In the current analysis at a median follow-up of 158 months, we report updated long-term results.

## 2 Methods

### 2.1 Patient selection and characteristics

From March 2004 to January 2008, 351 patients were treated with HDR monotherapy for clinically localised PCA receiving two BRT implants, separated by 14 days, each delivering two fractions at 9.5 Gy **[16]**. The present report describes the updated long-term results of our two-implant approach.

All patients had histologically proven PCA. Pre-treatment staging included digital rectal examination, transrectal ultrasound (TRUS), computed tomography (CT) of the pelvis/abdomen, pelvic magnetic resonance imaging (MRI) and bone scintigraphy. The Memorial Sloan Kettering group definition was used to classify patients into risk groups [16]. Eligibility criteria were clinically and radiographically organ-confined disease. In the case of high-risk disease, treatment was performed in men who rejected prostatectomy or EBRT, or who were not considered suitable for prostatectomy or definitive dose-escalated EBRT. Patients were considered ineligible for treatment in case of locoregional nodal or distant metastases, previous pelvic EBRT for another malignancy or previous surgery of the prostate.

Hormonal therapy was prescribed neoadjuvantly and continued concurrently with radiation and adjuvantly for an overall duration of median nine months (range, 3–14 months). The duration of ADT for the subgroups of low-risk, intermediate-risk, and high-risk patients was 4 months (range, 3–6 months), 6 months (range, 6–10 months) and 9 months (range, 9–14 months), respectively. Final decisions concerning hormonal treatment were made by the referring urologists. Patient and tumor characteristics of the updated analysis are shown in Table 1.

**Table 1:** Patient and tumor characteristics (*n* = 201).

Characteristics	
Median follow-up (months)	157.8 (20.3–199.7)
Gland volume at implant (cc)	
mean	42
median	39 (19–102)
Age at treatment (years)	
mean	67.2
median	67.6 (44.1–80.7)
Pre-treatment PSA (ng/ml)	
mean	6.9
median	6.2 (2.0–34.1)
	

	n (%)

Stage	
T1b-c	67 (33.3%)
T2a	61 (30.3%)
T2b	34 (16.9%)
T2c	39 (19.4%)
T3a	0 (0%)
Gleason score	
≤ 6	156 (77.6%)
7	39 (19.4%)
≥ 8	6 (2.9%)
Pre-treatment PSA (ng/ml)	
≤ 10	186 (92.5%)
11–20	13 (6.4%)
> 20	2 (0.9%)
Age at treatment (years)	
< 60	32 (15.9%)
60–69	100 (49.7%)
≥ 70	69 (34.3%)
Androgen deprivation therapy	62 (30.8%)
Risk group	
Low	103 (51.2%)
Intermediate	47 (23.3%)
High	51 (25.3%)

### 2.2 Brachytherapy protocol

A general description of our HDR technique has been published previously [16]. In brief, transperineal implantation was performed under TRUS guidance after inverse pre-planning using the intraoperative treatment planning system SWIFT (Nucletron B.V., Veenendaal/ELEKTA, Sweden). Evaluation of implant conformity was based on dose-volume parameters for the planning target volume (PTV, defined as the entire prostate gland without margins) and organs at risk (OAR, i.e. urethra and rectum). Dose specification was given as the mean dose on the PTV surface. The prescribed reference dose was 9.5 Gy delivered four times to a total dose of 38.0 Gy using two implants separated by 14 days, each delivering two treatment fractions with an interfraction interval of 6 h. Dosimetry assessment parameters and OAR constraints are shown in Table 2. All treatments were performed using an ^192^Iridium HDR afterloading system (microSelectron-HDR, ELEKTA, Sweden).

**Table 2:** Dosimetry parameters of the brachytherapy protocol with total physical prescription dose (Gy) and normal tissue dose constraints (as percentages of the prescribed reference dose).

Treatment scheme	PTV	D_10_/D _0.1 cm^3^_ Rectum	D_10_/D _0.1 cm^3^_ Bladder	D_10_/D _0.1 cm^3^_ Urethra	D90	V100	V150
9.5 Gy × 4	38.0 Gy	≤ 75% / ≤ 80%	≤ 75% / ≤ 80%	≤ 115% / ≤ 120%	≥100%	≥ 90%	≤ 35%

PTV = planning target volume

D10-Rectum = dose delivered to 10% of the rectum

D10-Bladder = dose delivered to 10% of the bladder

D10-Urethra = dose delivered to 10% of the urethra

D _0.1 cm^3^_ = minimum dose to the most exposed 0.1 cm^3^ of the organ

D90 = dose delivered to 90% of the PTV

V100, V150 = percentage of PTV receiving 100% and 150% of the prescription dose

### 2.3 Follow-up and data management

Initial follow-up was performed at six weeks after the last BRT fraction and then every three months for the first year, every six months for the second year and annually thereafter. Patient data was collected from a prospectively maintained database and by retrospective clinical chart review with data collection allowing also information acquisition from referring urologists. Toxicity analysis for GI and GU sites was performed using the National Cancer Institute Common Toxicity Criteria for Adverse Events, version 3. Toxicity was scored per event, and the highest value noted during follow-up was selected for statistical analysis. Biochemical relapse was defined according to the Phoenix definition [17]. Potency was defined as the ability to achieve an erection sufficient for intercourse.

All treated patients received in December 2019 as well as, in case of no response, in October 2020, a questionnaire to determine their PSA status and to assess whether the last documented adverse events were persistent or not. Questionnaires were returned in 162 cases until December 2020. In addition, we collected verified follow-up data for 39 additional patients from referring urologists and facility medical records. Overall, 201 patients were eligible for analysis in the current report. Out of the initial cohort, 62 patients died during follow-up and 150 were lost to follow-up. The median follow-up for the current analysis was 157.8 months compared to 59.3 months in the initial report.

### 2.4 Statistical analysis

The estimated likelihood of events was calculated using the Kaplan-Meier actuarial method and comparisons made using the log-rank test. A 2-sided *P* value of 0.05 was considered statistically significant. Biochemical control was calculated from the date of BRT to the date of biochemical failure or initiation of androgen deprivation for presumed biochemical relapse. Metastasis-free survival (MFS) was calculated from the date of BRT to the date when distant metastases were identified.

## 3 Results

### 3.1 Clinical outcome

Of the 201 patients analyzed, 26 (12.9%) experienced biochemical failure with 8 (3.9%) developing distant metastatic disease. The estimated BC was 94% at 60 months, 90% at 120 months and 86% at 158 months. The estimated overall survival (OS) was 94% at 60 months, 85% at 120 months and 74% at 158 months. The estimated MFS was 97% at 60 months, 89% at 120 months and 83% at 158 months.

**Fig. 1:**
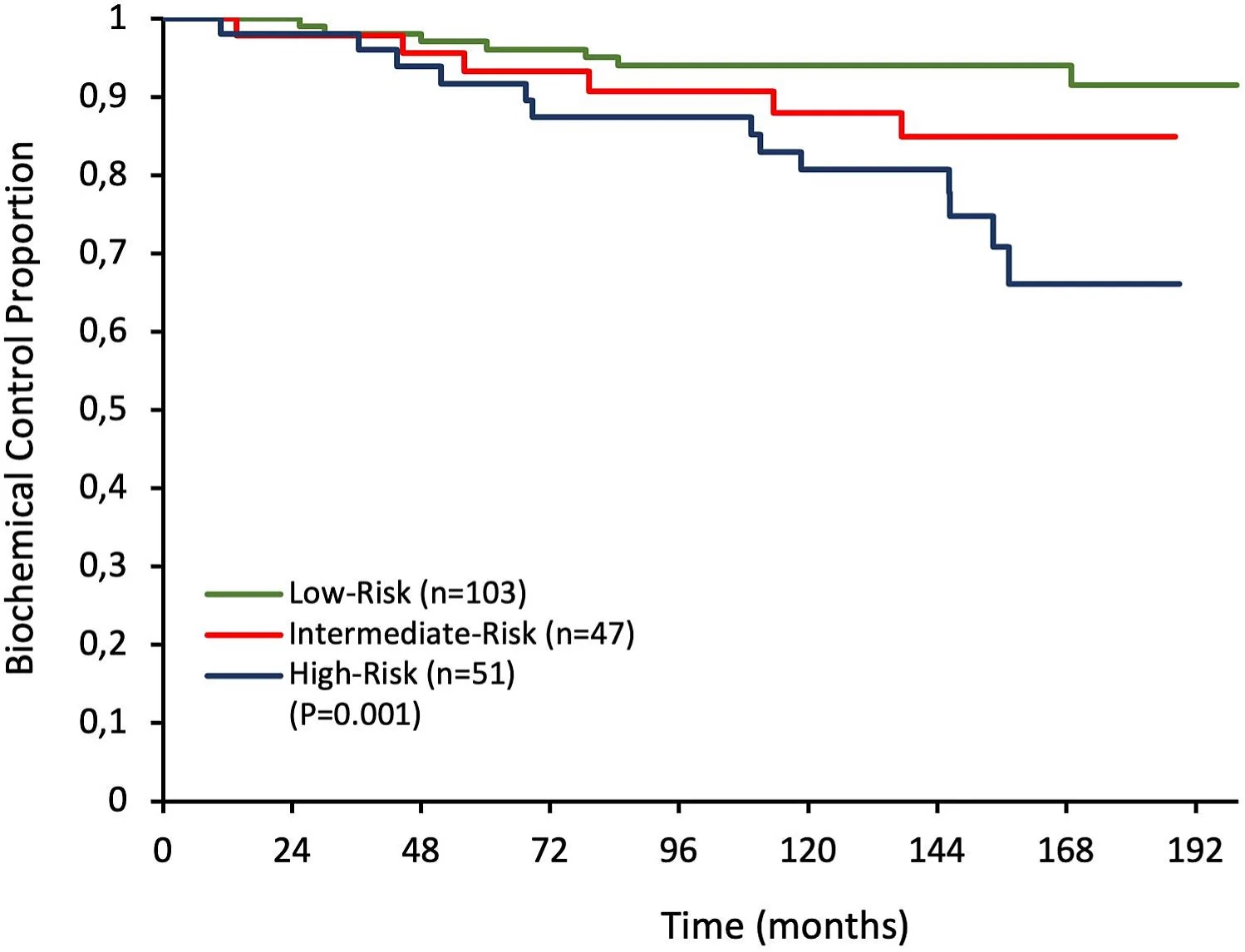
Kaplan-Meier actuarial analysis of all 201 patients for biochemical control by risk group.

The BC at 158 months according to risk group was 94%, 85%, and 71% for low-, intermediate- and high-risk patients, respectively, which did achieve statistical significance (*P* = 0.001, Fig. 1) The BC at 158 months according to Gleason Score (GS) was 90%, 75%, and 50% for patients with GS ≤ 6, 7, and ≥ 8, respectively (P = 0.001). This was statistically significant. The BC at 158 months according to clinical stage was 91%, 87%, and 68% for patients staged ≤ T2a, T2b, and ≥ T2c, respectively (*P* = 0.01). Again, there was a statistically significant difference. Biochemical control at 158 months for the subgroup of high-risk patients who received ADT was 73% and 67% for patients without ADT (*P* = 0.78). There was no statistically significant difference. The Kaplan-Meier estimates of BC at 158 months according to pre-treatment PSA were 88%, 66% and 100% for patients with a PSA of ≤10, 11–20, and > 20 ng/ml, respectively, and also did not achieve statistical significance (*P* = 0.10). Characteristics used for multiple regression analyses to correlate with BC were clinical T-stage (≤ T2a, T2b, ≥ T2c), GS (≤ 6, 7, ≥ 8), pre-treatment PSA (≤ 10, 11–20, > 20 ng/ml) and ADT. Multivariate Cox regression analyses could identify ADT as an independent prognostic factor for BC.

The median follow-up for the current analysis was 158 months with 198, 189, 170 and 100 patients reaching the 36, 60, 120, and 158 months survival time points, respectively.

### 3.2 Toxicity

Table 3 shows the results for late adverse events. Eight (3.9%) patients developed late Grade 2–3 lower urinary tract symptoms characterized by increased urgency and frequency with no > Grade 2 dysuria or incontinence. Four (2%) patients developed late Grade 3 GI toxicity, two of whom had endoscopically verified Grade 3 rectal mucositis. No Grade 4 or higher acute or late toxicities occurred. Erectile dysfunction (ED) Grade 3, defined as a medically assisted ED inadequate for intercourse, was reported by 14 (6.9%) patients prior to BRT. Of the 187 patients who reported Grade 0–2 ED prior to treatment, 27 (13.4%) confirmed a decline to Grade 3 after BRT.

**Table 3:** Late toxicity results (n = 201).

Toxicity	Grade			
	1	2	3	4
Genitourinary				
Frequency/Urgency	18 (8.9%)	6 (2.9%)	2 (0.9%)	–
Dysuria	11 (5.4%)	4 (1.9%)	0	0
Incontinence	13 (6.4%)	13 (6.4%)	0	0
Erectile Dysfunction	48 (23.8%)	29 (14.4%)	41 (20.3%)	–
Gastrointestinal (Rectum)				
Pain	6 (2.9%)	0	1 (0.4%)	0
Mucositis/Necrosis	0	2 (0.9%)	2 (0.9%)	0
Diarrhoea	0	0	0	0

## 4 Discussion

High-dose-rate monotherapy has proven to be an effective treatment modality for localised PCA with superior conformity compared to non-stereotactic EBRT techniques [14], [18]. In this study we expanded the follow-up of our previously reported-on cohort [16] to a median of 158 months. After correcting for patients who were lost to follow up, 26 biochemical relapses (12.9%) and 8 metastatic events (3.9%) were documented, generating an actuarial BC of 86% at a median follow-up of 158 months. This oncologic outcome is in line with other published HDR monotherapy data overseeing follow-up periods equal to or longer than 60 months [19], [20], [21], [22], [23], [24], [25], [26], [27], [28], [29].

In our updated analysis encompassing 201 patients, characteristics used for multiple regression analyses to correlate with BC were clinical T-stage, GS, pre-treatment PSA and ADT. The multivariate multivariate Cox regression analyses could identify ADT as an independent prognostic factor for BC. Rogers et al. [30], Hoskin et al. [31] and Yoshioka et al. [32] likewise failed to verify GS and pre-treatment PSA or clinical T-stage as significant predictors of risk for biochemical recurrence. Hoskin et al. [31] reported on a group of 197 patients with a four-year BC rate of 87% in 86 high risk-cases. Those included clinical stages ≥ T3 in 21%, GS ≥ 8 in 10%, and PSA > 20 in 25% of cases with 92% of high-risk patients receiving temporary ADT.

In our study, the BC at 158 months for low-risk, intermediate-risk and high-risk patients was 94%, 85% and 71%, respectively. There was a statistically significant difference between these risk groups indicating a superior benefit for low- and intermediate risk patients compared to the high-risk collective. At this, 62.7% of high-risk patients received temporary ADT including all patients with PSA ≥ 20 ng/ml, all patients with GS ≥ 7b (4 + 3), and 53.8% of all cases staged > T2b. The advantage of short-term androgen suppression for high-risk disease, however, remains a topic of ongoing discussion as there is no prospective evidence supporting it in clinically non-metastatic patients receiving HDR BRT. In this context, the Kaplan-Meier actuarial estimated of BC at 158 months for the subgroup of high-risk patients in our series who received ADT was 73% and 67% for patients without ADT, (*P* = 0.78). There was no statistically significant difference. Notwithstanding, HDR monotherapy for high-risk disease can be questioned given the factual risk for outcome-driving extraprostatic disease spread. In our cohort, none of the high-risk patients was systematically staged with prostate-specific membrane antigen (PSMA) positron emission tomography (PET)/CT and pelvic MRI in the setting of cT3/GS ≥ 8/PSA > 20 ng/ml – disease.

Our analysis confirmed low rates of late GI- and GU-toxicity corroborating the safety of HDR monotherapy as demonstrated by published long-term experiences of other groups [19], [20], [21], [22], [23], [24], [25], [26], [27], [28], [33].

Eight (3.9%) patients developed late Grade 2–3 lower urinary tract symptoms characterized by increased urgency and frequency with no > Grade 2 dysuria or incontinence. Four (2%) patients developed late Grade 3 GI toxicity, two of whom had endoscopically verified Grade 3 rectal mucositis with no Grade 4 acute or late adverse events documented. Erectile dysfunction Grade 3 increased in this update from 16.5% to overall 20.3%, consisting of all patients with pre-existing as well as declined function, which is not unexpected given the median patient age of 67.7 years for the analyzed cohort during treatment in 2007–2009. Notwithstanding, almost 80% of patients self-reported an erectile function suitable for intercourse, with or without the use of medical aids, as calculated by the questionnaire-based analysis. This outcome is consistent with published experiences for the same age group, showing that ED is infrequently observed [25], [28], [30], [34], [35], [36], [37], [38], [39], [40] and solidifies the safe side effect profile of HDR monotherapy. In addition, contrasting juxtaposition of HDR and LDR monotherapy data have recurrently shown reduced rates of both acute and late high-grade toxicities after HDR, making it the more innocuous of the two modalities [14]. Furthermore, extreme hypofractionation as the status quo in BRT is backed by the current scientific knowledge in radiobiology. Strouthos et al. [14] aggregated data, exhibiting feasible mean biologically effective dose (BED) values of 256 Gy (*α*/*β-*ratio of 1.5 Gy) and EQ.D2 doses ranging from 89 to 128 Gy through HDR, values hardly attainable using EBRT as of today.

Limitations of our analysis are firstly the retrospective design. We conducted this study without a control group. While 62 patients passed away due to causes not associated with PCA progress, 150 were lost to follow-up and possibly skewing BC and rate of metastasis. In addition, there was non-uniform initiated ADT based on the discretion of the referring urologists. Nevertheless, the present analysis is bolstering data supporting HDR monotherapy for low- and intermediate risk PCA as safe and efficient treatment expanding well beyond the 120-month follow-up period**.**

## 5 Conclusion

Our mature follow-up data corroborate sole interstitial HDR radiotherapy to be an effective treatment option for localised PCA, while entailing few side-effects, independent of risk group stratification. For tumor modalities exceeding aforementioned categories, however, explorative investigations are needed.

## CRediT authorship contribution statement

**Christopher Best:** Writing – review & editing, Writing – original draft, Software, Formal analysis, Data curation. **Valentin Spier:** Writing – review & editing, Data curation. **Dimos Baltas:** Writing – review & editing, Methodology, Investigation, Data curation. **Hanns Ackermann:** Writing – review & editing, Formal analysis. **Claus Rödel:** Writing – review & editing. **Nikolaos Zamboglou:** Writing – review & editing, Validation. **Nikolaos Tselis:** Writing – review & editing, Writing – original draft, Validation, Supervision, Methodology, Investigation, Data curation, Conceptualization.

## Funding

No funding was received for this study.

## Declaration of competing interest

The authors declare that they have no known competing financial interests or personal relationships that could have appeared to influence the work reported in this paper.
